# Pleomorphic Adenoma of a Minor Salivary Gland of the Hard Palate: A Case Report

**DOI:** 10.7759/cureus.47957

**Published:** 2023-10-30

**Authors:** Ishank Panchal, Anil Wanjari

**Affiliations:** 1 Medicine, Jawaharlal Nehru Medical College, Datta Meghe Institute of Higher Education and Research, Wardha, IND

**Keywords:** salivary gland, report, case, adenoma, pleomorphic

## Abstract

Pleomorphic adenoma affects mostly the parotid gland (85%), submandibular glands (5%), and the minor salivary glands (5%). They are usually referred to as benign mixed tumors because they are composed of both epithelial cells, which are cells from the body's surface, and myoepithelial cells, present inside glands to aid in secretion. An 88-year-old male who had an ulcer for 10 days and a three to four-month-old swelling on his palate arrived in the OPD. On examination, at the intersection of the hard and soft palates, there is a single, well-delineated, nodular exophytic development of small salivary glands left to the median half (most of the pleomorphic adenomas are unilateral). For further and confirmatory diagnosis, fine needle aspiration cytology, immunohistochemistry tests, and radiodiagnosis, i.e., X-rays, were performed which revealed periapical abscess and swelling on palate unassociated with pus discharge. Surgical removal of the swelling and extraction of the root stump was done. The patient was asked to stop smoking strictly. Post-operatively, the patient was given suitable medications.

## Introduction

The most frequent salivary gland tumor, pleomorphic adenoma, is sometimes referred to as a benign mixed tumor due to its dual genesis from myoepithelial and epithelial components [1]. It makes up two-thirds of all salivary gland tumors, making it the most prevalent [2,3]. Pleomorphic adenomas, the most common type of tumor, make up about 66% of all salivary gland neoplasms [4]. Willis coined the phrase "pleomorphic adenoma" first [5]. It was also known as a mixed tumor, enclavoma, branchioma, endothelioma, endochroma, etc., in former times [6]. All salivary glands originate from oral epithelial ingrowths, with the parotid anlage beginning to form between four and six weeks after formation. The absence of lymphoid tissue in the latter is explained by the fact that lymphoid tissue develops after the encapsulation of the submandibular and sublingual glands but before the parotid gland. The incidence is 85% in the parotid glands, 10% in the minor salivary glands, and 5% in the submandibular glands [7]. Most adult females with pleomorphic adenoma are in their third to fifth decades of life. According to the World Health Organization, pleomorphic adenoma is a confined tumor, has pleomorphic or mixed epithelial origin characteristics, and is interspersed with mucoid tissue, myxoid tissue, and chondroid masses. It accounts for 66-90% of all salivary gland tumors in children, making it the most typical salivary gland neoplasm [8]. The preferred course of therapy is wide local excision with removal of the periosteum and affected bone [9,10]. There is a 6% chance that the pleomorphic adenoma may develop into cancer. If not surgically removed, they can degenerate into carcinomas. Once they turn carcinomatous, they are named carcinoma ex pleomorphic adenoma (CXPA). Pleomorphic adenoma is usually asymptomatic, except for the tendency to grow to reach significant dimensions. This is why symptoms usually depend on the volume reached by the mass and the structures that are consequently compressed. Pleomorphic adenoma tumors are not painful, have clear borders, and are covered with healthy mucous membranes. Ulcerations of the mucosa can occasionally be seen. Single and movable related nodules are seen. Unlike small gland tumors, primary gland tumors are typically encapsulated.

A few pathological characteristics, such as invasiveness, have also been studied; extracapsular extension and invasion of less than 1.5 mm predict greater mortality and recurrence [11]. Although prior studies have focused on the molecular and pathophysiological elements of CXPA of the main and minor salivary glands, a thorough evaluation of clinical therapy, prognostic variables, and long-term follow-up outcomes has not yet been conducted [12].

## Case presentation

An 88-year-old male patient came with a chief complaint of swelling on the palate for three to four months with an ulcer for 10 days. Swelling was present left to the median half of the hard palate. It gradually increased its size and was not associated with any pus discharge. History dates back to four months ago when the patient first experienced swelling present left to the median half of the hard palate. Swelling was not associated with pain and gradually increased in size. Ten days ago, a single ulcer was observed associated with a lesion about 1 cm in size and irregular shape. There was no relevant past dental and medical history. The patient has smoked 15 bidis daily for the last 70 years.

**Figure 1:**
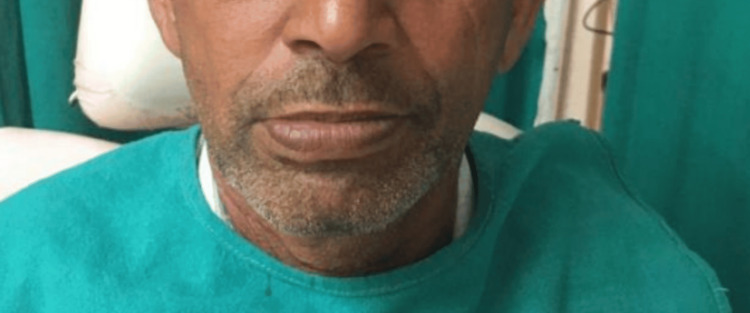
Front profile of the patient with facial symmetry and no extra-oral lesion

During the clinical examination, no significant or relevant extra-oral findings were present (Figure 1).

On intra-oral examination, swelling of about 1 inch in diameter with ulceration of 1 cm in irregular shape left to median half on the hard palate was observed.

**Figure 2:**
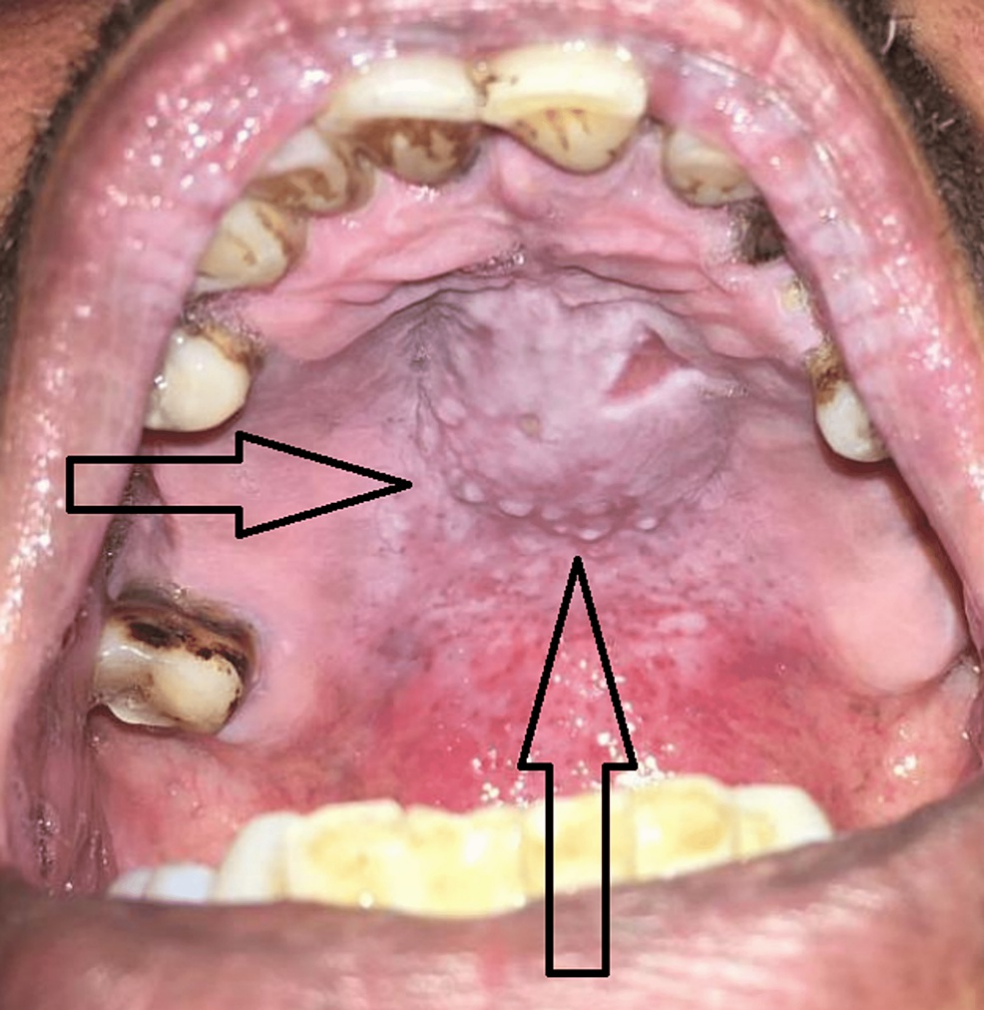
Solitary, well-defined nodular exophytic growth originating from the minor salivary glands on the left side of the median half of the hard palate, measuring approximately 1 inch in diameter. The lesion's surface epithelium protrudes, resulting in an irregularly shaped ulcerated surface of about 1 cm in size with a sloping edge

**Figure 3:**
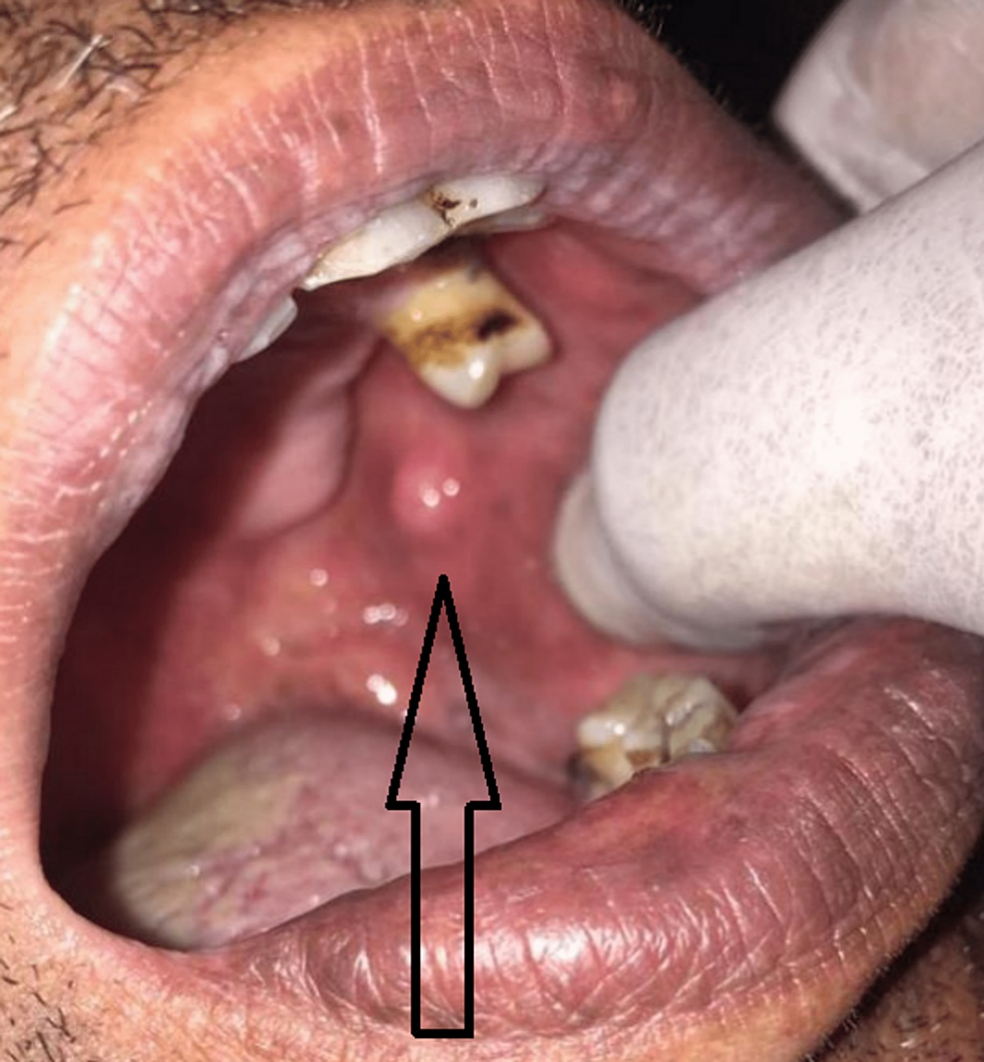
Soft tissue overgrowth measuring 0.5 cm in size, located in the molar region on the left side

On local examination/examination of the lesion, a solitary, well-delineated, nodular exophytic growth of minor salivary glands left to median half on the hard was present, which was about 1 inch in diameter. The swelling was not associated with pus discharge. The surface epithelium of the lesion burst out to give an ulcerated surface of about 1 cm in size, which was irregular in shape and had a sloping edge associated with mild pain (Figures 2-3), off and on that began gradually and was continuous in nature which was non-radiating. Pain aggravates on touch and relieves on its own after some time. All inspection findings were confirmed on palpation. The lesion exhibited a firm consistency, was unilocular, and had well-defined margins. It was immovable. The mucosa over the lesion was stretched and non-pinchable. The ulcer did not bleed on palpation.

**Figure 4:**
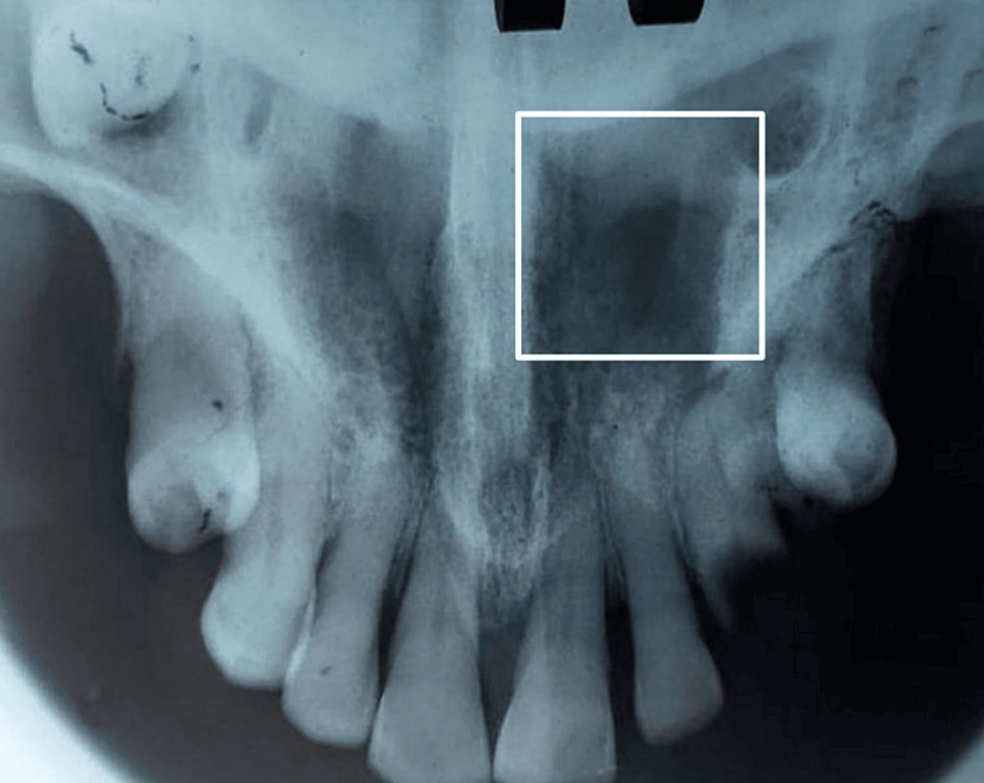
Ill-defined radiolucency at 23 and 24 showing the abscess

On radiological examination, ill-defined radiolucency at 23, 24 with abscess (Figure 4), and intra-oral periapical at root stump 34 was seen.

Following the findings, the patient was informed of his condition and urged to have surgery to completely remove the cyst, which was done successfully.

Operative notes

This case was referred to an oral surgeon. The pleomorphic adenoma was successfully removed surgically with sufficient margins, and root stump extraction was also done.

The surgery performed was hemimaxillectomy with the reconstruction of the temporalis muscle flap. It was performed under general anesthesia. Submental intubation was done as nasal intubation was not possible. No post-operative complications were there. A complete cessation of smoking was advised.

## Discussion

Pleomorphic adenoma is a complex morphological epithelial tumor that is embedded in a mucopolysaccharide stroma and has both epithelium and myoepithelial components organized in different ways. The surrounding salivary parenchyma, which the tumor compresses and causes to fibrose, is what causes the "false capsule" to form.

Pleomorphic adenoma of the minor salivary gland of the hard palate is a rare occurrence. It accounts for 5-7% of cases among the various salivary gland tumors. In pathology laboratories, small salivary gland tumors are thought to account for 0.35-1.5% of all biopsies. The palate lip, nasal cavity, pharynx, larynx, and trachea are the most often occurring locations. It usually presents with slow-growing, painless, well-delineated, and nodular exophytic growth. Depending on the location of the tumor, minor salivary gland tumors can cause a range of symptoms, such as dysphagia, hoarseness, dyspnea, trouble chewing, and epistaxis.

Finding the right diagnosis can be tough. Fine needle aspiration cytology, MRI, immunohistochemistry, and special stains are used to make the diagnosis. Surgery is the primary form of therapy and has a very good cure rate. High-grade disease, cases of uncertain resection adequacy, lymph nodes, and peri-neural invasion are treated with post-operative radiation. Additionally, patients may be given the choice of receiving both radiation and chemotherapy [13]. On the efficacy of chemotherapy in the treatment of CXPA, there is, however, little research [14]. Patients with positive margins, inoperable tumors, and multifocal recurrences following earlier resection might benefit from radiotherapy (RT) to achieve local control. For microscopic and gross residual tumors, the local control rates with RT are around 80% to 85% and 40% to 60%, respectively [15]. Treatment factors for pleomorphic adenoma of both major and minor salivary glands is an area to be researched further.

## Conclusions

The patient came with complaints of swelling without pus accompanied by pain. The lesion had a firm consistency, was unilocular, and had well-defined margins. It was also immovable. The mucosa over the lesion was stretched and non-pinchable with a non-bleeding ulcer. All these findings were suggestive of pleomorphic adenoma and chronic generalized periodontitis, and it was confirmed pathologically and through other tests. A surgical hemimaxillectomy with reconstruction of the temporalis muscle was done, and there were no complications post-operative. The patient was given medications and was advised not to smoke ever again.
